# Identifying the Molecular Drivers of Pathogenic Aldehyde Dehydrogenase Missense Mutations in Cancer and Non-Cancer Diseases

**DOI:** 10.3390/ijms241210157

**Published:** 2023-06-15

**Authors:** Dana Jessen-Howard, Qisheng Pan, David B. Ascher

**Affiliations:** 1 School of Chemistry and Molecular Bioscience, University of Queensland, Brisbane, QLD 4072, Australia; d.jessenhoward@uq.net.au (D.J.-H.); qisheng.pan@uq.net.au (Q.P.); 2 Computational Biology and Clinical Informatics, Baker Heart and Diabetes Institute, Melbourne, VIC 3004, Australia

**Keywords:** aldehyde dehydrogenase, cancer, missense mutations, machine learning, pathogenic molecular driver

## Abstract

Human aldehyde dehydrogenases (ALDHs) comprising 19 isoenzymes play a vital role on both endogenous and exogenous aldehyde metabolism. This NAD(P)-dependent catalytic process relies on the intact structural and functional activity of the cofactor binding, substrate interaction, and the oligomerization of ALDHs. Disruptions on the activity of ALDHs, however, could result in the accumulation of cytotoxic aldehydes, which have been linked with a wide range of diseases, including both cancers as well as neurological and developmental disorders. In our previous works, we have successfully characterised the structure–function relationships of the missense variants of other proteins. We, therefore, applied a similar analysis pipeline to identify potential molecular drivers of pathogenic ALDH missense mutations. Variants data were first carefully curated and labelled as cancer-risk, non-cancer diseases, and benign. We then leveraged various computational biophysical methods to describe the changes caused by missense mutations, informing a bias of detrimental mutations with destabilising effects. Cooperating with these insights, several machine learning approaches were further utilised to investigate the combination of features, revealing the necessity of the conservation of ALDHs. Our work aims to provide important biological perspectives on pathogenic consequences of missense mutations of ALDHs, which could be invaluable resources in the development of cancer treatment.

## 1. Introduction

**Figure 1:**
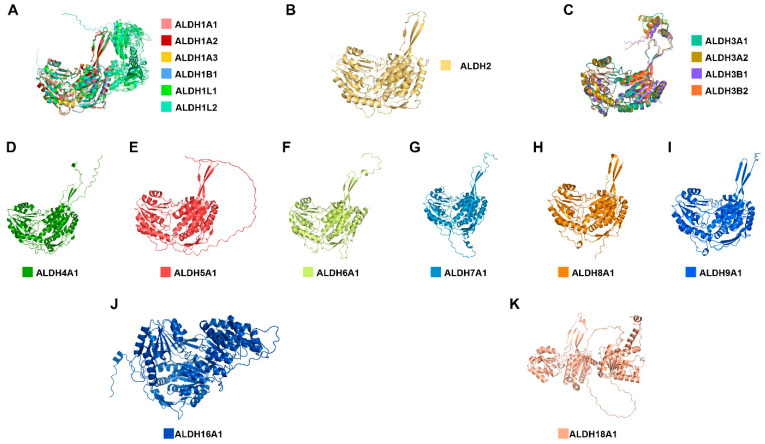
Protein structures predicted by AlphaFold2 of human ALDHs. Each ALDH was assigned a colour, which was grouped into 11 different families, namely ALDH1 (**A**), ALDH2 (**B**), ALDH3 (**C**), ALDH4 (**D**), ALDH5 (**E**), ALDH6 (**F**), ALDH7 (**G**), ALDH8 (**H**), ALDH9 (**I**), ALDH16 (**J**), and ALDH18 (**K**). Human ALDHs share similar folding on cofactor binding and substrate binding regions, particularly found in the ALDH1 and ALDH3 family, while some ALDHs (ALDH1L1, ALDH1L2, ALDH16A1, and ALDH18A1) contain additional domains performing other cellular functions.

Aldehyde dehydrogenases (ALDHs) are part of an enzyme superfamily that interacts with endogenous and exogenous aldehyde metabolism. The human ALDH family comprises 19 isotypes that share common structural and functional features, such as the similar folding of cofactor binding and substrate binding regions (Figure 1) [1], with over 40% sequence identity (Figure S1), but they are involved with a large variety of cellular processes [2]. ALDHs utilise Nicotinamide adenine dinucleotide phosphate (NAD(P))-dependent reactions to catalyse the conversion of aldehydes to carboxylic acids, including in the detoxification of alcohol-derived acetaldehyde and synthesis of retinoic acid.

Human ALDHs are composed of monomeric, dimeric, tetrameric, and octameric structures containing a catalytic domain with a conserved Cysteine active site located at the N-terminal region and responsible for catalytic activity, a cofactor domain which is responsible for binding to coenzyme at the C-terminal region [3], and an “arm-like” oligomerization domain [4]. The catalytic domain is constructed by 10–12 alpha-helices and a few beta-strands and is where the oxidation reaction takes place, whereas the cofactor domain is composed of several beta-strands and forms a Rossmann fold [5]. Some ALDHs, such as ALDH1L1, ALDH1L2, and ALDH18A1, can have other domains, including regulatory, dimerization, or signal peptide features (Figure 1). Oligomerization mechanisms are essential in the maintenance of proper ALDH function and disease-free states [4].

The disruption of ALDH activity has been linked to a wide range of diseases including epilepsy [6,7], alcohol liver disease [8], sjorgen-larrson syndrome [9], hyperprolinemia [10], hyperammonemia [11,12], and aciduria [13]. The role of ALDHs in cancer, however, is more complex. Accumulation of damage from carcinogenic aldehydes, particularly in tobacco smoke and alcohol, has been linked to the development of different types of tumours. But interestingly, the subsequent increase in ALDH activity within cancers has been associated with a poorer cancer prognosis/recovery, and worse chemotherapy treatment outcomes [2]. This double-edged role in cancer is well illustrated by mutations in ALDH2, which can act as a tumour suppressor by reducing the damaging effects of aldehydes and as oncogenic through drug detoxification and promotion of cell survival/growth [14,15]. These associations are further complicated by incomplete penetrance, with, for example, high levels of ALDH1 expression not always strongly correlated with phenotypes of high malignancy and poor patient outcomes [15].

This complexity has hindered efforts to rationally and systematically characterise the role of ALDH mutations in diseases. We have previously shown that computational tools can be used to understand the consequences of missense mutations on protein structure, providing insight into molecular mechanisms of disease and further predicting disease outcome [16,17,18]. Towards better understanding the molecular consequences of disease-associated ALDH mutations, here, we have curated a set of high-confidence clinically observed missense mutations across the human ALDH genes, with particular validity in 16 of the 19 genes and characterising the effects of disease-causing missense mutations on protein structure and function in order to gain insights into major disease mechanisms.

## 2. Results

### 2.1. Genetic Variants with Different Biological Consequences in Human ALDHs

**Figure 2:**
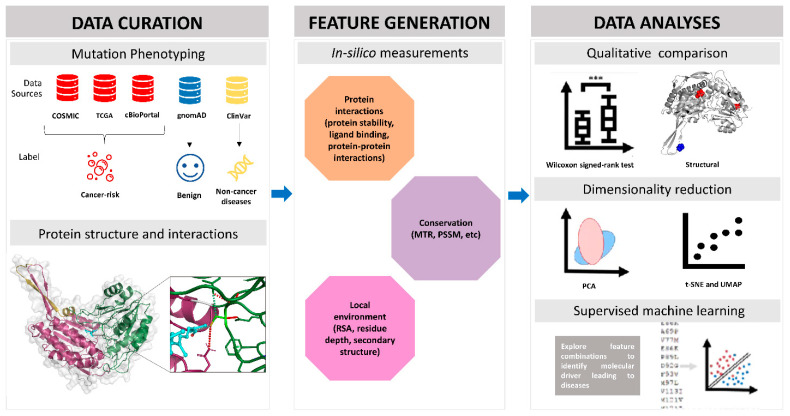
Mutation analysis pipeline to identify pathogenic molecular drivers of ALDHs. This workflow consists of three steps. Missense variants were first curated from multiple databases and labelled as cancer-risk, benign, and non-cancer diseases, respectively. ALDH protein bound with substrates was generated by AlphaFold2 and AutoDock Vina. After this, various computational biophysical measurements were used to annotate the missense mutations on different aspects such as protein interactions, conservation, and local residue environment. Lastly, we implemented both qualitative analysis and machine learning approaches to identify potential disease-causing risk factors.

The workflow we proposed to characterise the structural and functional consequences of ALDH missense mutations and their relationship with diseases is depicted in more detail in Figure 2. Genetic variant data for the 19 ALDH genes was explored through three cancer-associated databases, gnomAD, and ClinVar to reveal information for three different phenotypic consequences: cancer-risk, benign, and non-cancer diseases, respectively.

Missense variant data from three cancer-specific databases COSMIC, TCGA, and cBioPortal were leveraged to study the variants which present contributions to tumour development. After removing the duplicates and mismatched samples of canonical sequences, we initially collected 2822 unique mutations. There were 40 mutations across 13 ALDH proteins in our final cancer-risk group by taking overlap mutations with strong association (Table 1). These cancerous mutations are mainly related to adenocarcinoma in different tissues (35%) and cutaneous melanoma (35%), reflecting the heterogenicity of cancer. Due to the limited amount of data, we merged all these mutations related to different tumours into the same labels. Cancer-risk mutations were not found in all ALDHs, with 40% of these mutations located in the ALDH1 family alone.

In terms of non-cancer diseases, 817 data points of ALDH mutations were extracted from ClinVar databases, and only 38 of them came with clinical evidence of pathogenicity after literature validation (Table 1). Nine mutations found in ALDH7A1 show verified association with epilepsy-related conditions, while 11 mutations found in ALDH18A1 are confirmed with hereditary spastic paraplegia. The rest of the mutations associated with developmental disorders such as anophthalmia/microphthalmia and various heart and lung defects were sporadically distributed across other ALDHs.

As for variants we labelled as benign, there are 3932 entries in gnomAD databases, but after data cleaning, only 30 of them have allele frequency over 1%, which are distributed across 13 human ALDHs. Three mutations found in ClinVar were also considered benign following literature validation, which was already defined in these 30 common variants in populations. This overlap further improved the reliability of the labels of benign mutations.

### 2.2. Exploring the Distribution of Genetic Variants in Human ALDHs

**Figure 3:**
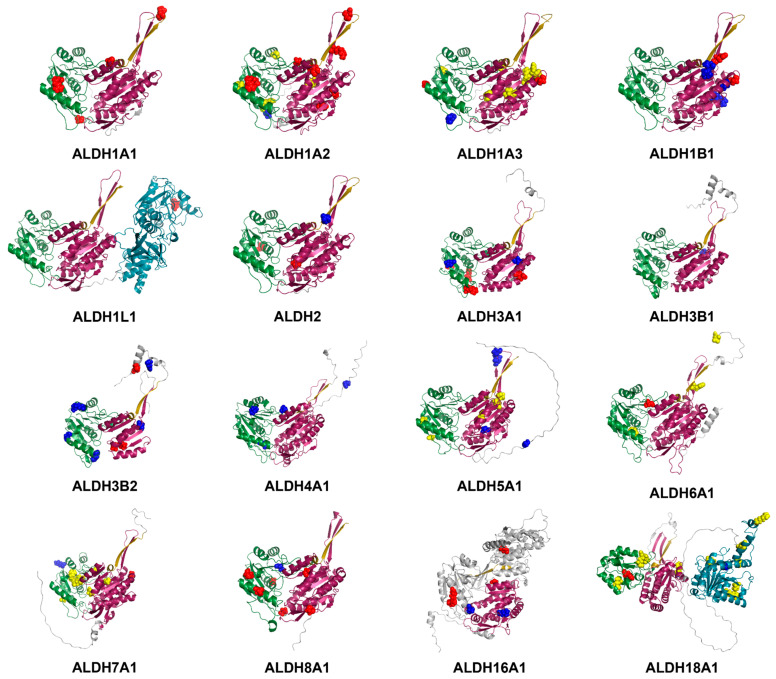
Distributions of variants of three labels, namely cancer-risk (red), benign (blue), and non-cancer diseases (yellow) of the structures of human ALDHs. Each ALDH protein is coloured based on its different important regions, namely NAD(P)+ binding region (dark magenta), aldehyde binding region (dark green), protein–protein interaction region (dark yellow), and addition domains such as folate/glutamate binding region (dark cyan).

In terms of the location in the sequences and structures of ALDHs, genetic variants labelled as benign, cancer-risk, and non-cancer diseases distribute sparsely in two major substrate binding regions, the NAD(P)+ binding site and the aldehyde binding site, while some missense mutations are found in the protein–protein interaction (PPI) region (Figure 3 and Figure S2). Two cancer-risk mutations found in ALDH1A1 (R395H) and ALDH1A2 (R412W) show a similar structural location at the NAD(P)+ binding region. Similar structural distribution can be found in S86L of ALDH1A3 and S91F of ALDH1B1. As described above (Table 1), most non-cancer diseases mutations are found in ALDH7A1 and ALDH18A1. Three non-cancer pathogenic mutations cluster at Glu427 (aldehyde binding region) of ALDH7A1 and two mutations with the same labels are located at Arg138 (glutamate binding region) of ALDH18A1. These genetic variants with different labels, however, have no strong correlation with these functional regions, implying that the potential pathogenic mechanism may not be strongly related to the change of substrate binding or the alteration of the interaction between monomers.

### 2.3. Exploring the Molecular Drivers of ALDH Mutations Leading to Diseases

We then tried to identify potential pathogenic molecular drivers by using a range of in silico methods of biophysical calculations. In particular, we explored three primary biophysical properties (protein stability, protein-substrate binding, and protein–protein binding) and the residue environment at the mutation site.

(a) Protein stability

Compared with the change of protein stability caused by benign mutations, the impact on protein stability caused by both cancer-risk and non-cancer pathogenic mutations tends to scale larger and presented as a larger extent of the ΔΔG values. This may indicate that a stronger stabilising/destabilising effect is not suitable for the correct function of ALDHs. Cancer-risk mutations have a similar distribution of the ΔΔG values with the benign mutations, which are also suggested by the non-significant difference. On the other hand, the non-cancer pathogenic mutations show a different pattern of these measurements. The ΔΔG values on these non-cancer pathogenic variants computed by four tools (SDM, DUET, DDMut, and SAAFEC-SEQ) are significantly lower than the ones of benign samples (Figure S3), consistent with their buried location in the protein structure (Figure S6). We believed that the destabilising effect caused by these pathogenic mutations could be a potential driver towards diseases of human ALDHs.

(b) The binding of ALDH with NAD+ and aldehyde

The effect of the mutations on the binding affinity of ALDH for NAD+ and aldehyde substrate was measured using mmCSM-Lig. Interestingly, we did not observe a significant difference between the benign and pathogenic mutations. Further to that, the effects of all mutations on substrate binding were relatively mild (|ΔΔG| < 1 Kcal/mol) (Figure S4), which is perhaps not surprising as most mutations were located over 10 Å away from the ligands. The cancer-risk mutations showed a stronger impact on the ALDH-substrate binding, especially on the interaction between ALDH and the aldehyde molecules. By contrast, variants leading to non-cancer diseases showed an increase in aldehyde binding affinity, implying some mechanistic differences to the cancer-risk mutations.

(c) Protein–protein interaction of ALDH dimer

Similarly, there was no significant difference in the binding affinity of the ALDH dimer (Figure S5). We noticed that the non-cancer pathogenic mutations tended to be located closer to the PPI interface and were also linked to a smaller predicted impact on the dimeric structure. As for the cancer-risk groups, these mutations showed a stronger destabilising effect on the interaction of monomers in the ALDH complex, according to the predictions of mCSM-PPI1.

(d) Residue environment

There were some interesting differences in the solvent accessibility of the mutation loci between the non-cancer disease and benign mutations, illustrated by both RSA and residue depth (Figure S6). Similar trends were also noticeable in the cancer-risk samples, but the difference did not reach the significance threshold. We did not observe significant differences in the mutation locations including secondary structure elements and dihedral angles.

Mutation tolerance was measured using the MTR score, a measure of purifying selection, which showed significant differences between both cancer and non-cancer pathogenic mutation and their benign counterparts. Both of these pathogenic residue mutations were intolerant, with lower MTR scores compared to the benign variants. This suggests that population-based measures could be a useful measure of pathogenicity in ALDHs.

### 2.4. Using the Structural Consequences of ALDH Mutations to Distinguish Distinct Disease Outcomes

This structural analysis identified a number of key molecular drivers distinguishing benign and pathogenic mutations in ALDHs. We, therefore, proposed to leverage these insights within both unsupervised and supervised machine learning architectures to further explore the relationship between biochemical and functional features and the disease-causing mutations.

(a) Dimensionality reduction

**Figure 4:**
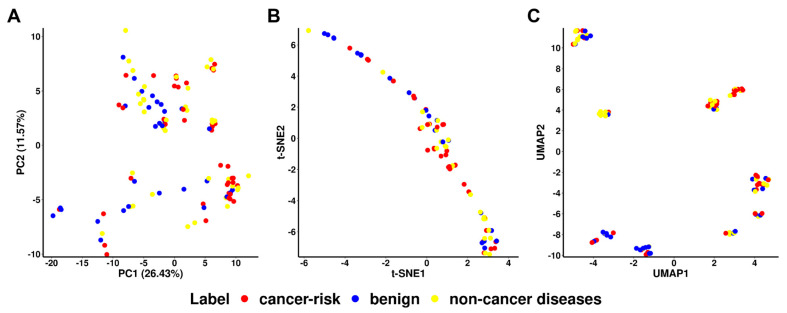
Visualisation of variants of three labels, namely cancer-risk (red), benign (blue), and non-cancer diseases (yellow) using dimensionality reduction methods, including Principal Component Analysis (PCA) (**A**), t-distributed stochastic neighbour embedding (t-SNE) (**B**) and Uniform Manifold Approximation and Projection (UMAP) (**C**), respectively. Data points were coloured based on their labels.

While different clustering approaches revealed the presence of some interesting distinct distributions of cancer and non-cancer pathogenic variants compared with the benign in a low dimensional space, the overall predicted power was only modest. Interestingly, it was actually the cancer-risk mutations that were more tightly clustered together than the mutations linked to other diseases (Figure 4). Benign variants were harder to differentiate, reflecting the overall challenge to accurately identify them.

(b) Model to identify cancer-risk variants

We trained a machine learning model to predict cancer-risk variants from benign and other-disease variants using the Gradient Boosting algorithm (Table 2). Due to the relatively small dataset, we used a bootstrapped 10-CV, which showed reasonable predictive performance, with an average Matthews Correlation Coefficient (MCC) up to 0.558. Both the assessments of Jack-Knife leave one protein out (LOPO) and showed a highly comparable performance with the results of 10-CV, providing confidence in the generalisability of our models. Compared with the state-of-the-art methods (Table 2, Figure S7), our cancer-risk model achieved similar performance in the classification of pathogenic mutations. Our model demonstrated a higher precision compared to the performance of Envision, indicating that it was able to more accurately predict cancer-risk variants.

**Figure 5:**
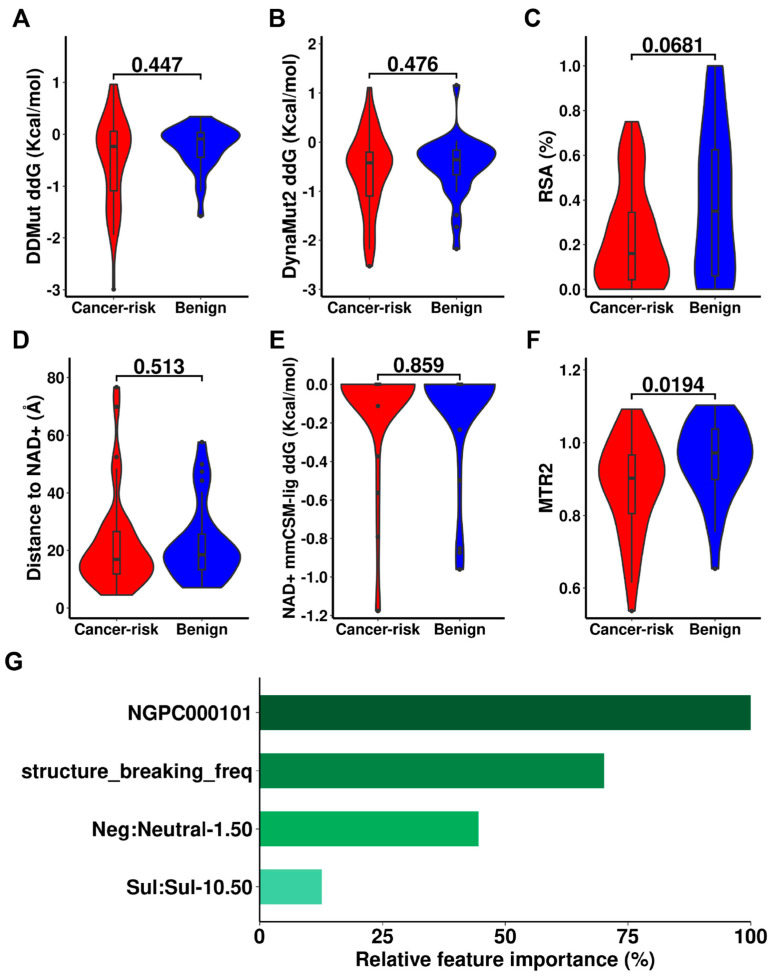
Potential molecular drivers leading to cancer of human ALDHs. Qualitative tests were performed using Wilcoxon signed-rank test on change of protein stability (**A**,**B**), Relative solvent accessibility (**C**), distance from mutation site to NAD+ (**D**), change of NAD+ binding affinity (**E**), and the Mutation Tolerance Ratio 2 (MTR2) (**F**) and between cancer-risk and benign mutations. Relative feature importance of the cancer-risk machine learning model was presented (**G**).

The model was interrogated to evaluate the contributions of each feature in order to reveal potential biological insight. Four features were selected in the optimisation process including a biological score NGPC000101 [19], the neighbour frequency of structure-breaking amino acid (G and P), and two atomic pairs patterns from the graph-based signatures (Figure 5). These features mainly refer to the biochemical environment of the mutation site, such as hydrophobicity and the specific amino acid composition formed by Glycine and Proline, and these are instrumental to the decision of risk of cancer.

We applied our cancer-risk model on the 2822 data points initially curated from three cancer databases for further experimental verification, which is available in the Supplementary Files.

(c) Model to identify non-cancer diseases variants

Similarly, we trained an Adaptive Boosting-based model to predict non-cancer pathogenic variants. Our final model demonstrated strong predictive performance across bootstrapped 10CV, LOPO, and Jack-Knife validation with MCCs up to 0.765, and outperforming all the other variant effect predictors (Table 3). The capability to classify non-cancer disease-causing mutations is slightly stronger than the one to identify cancer-risk mutations presented by the higher performance on the non-cancer diseases model, which is consistent with the findings in the qualitative test.

**Figure 6:**
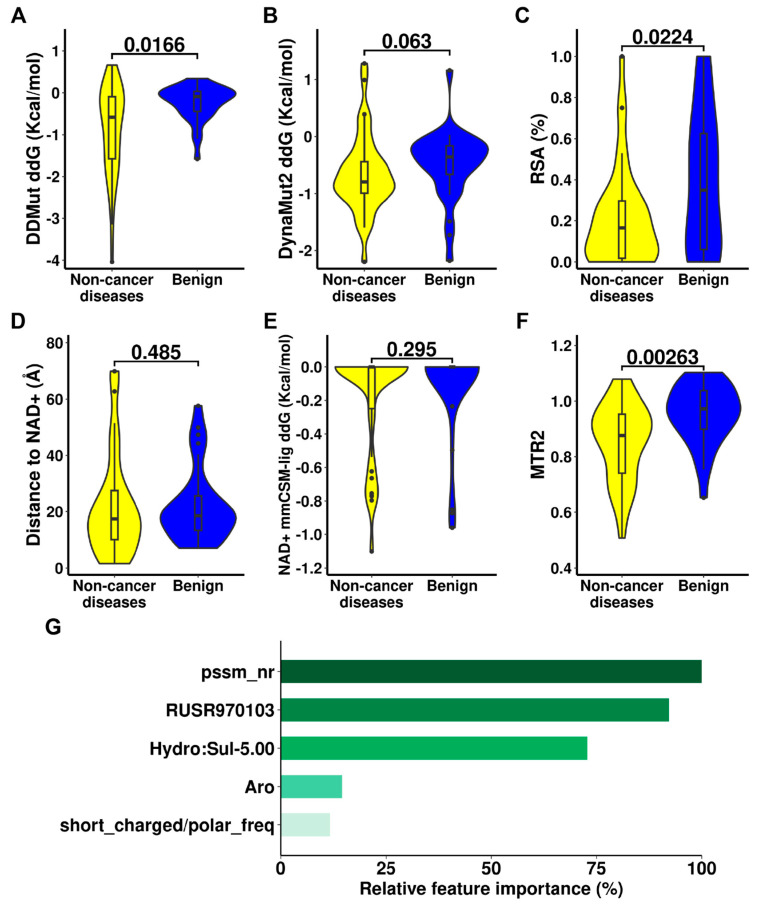
Potential molecular drivers leading to non-cancer diseases of human ALDHs. Qualitative tests were performed using Wilcoxon signed-rank test on change of protein stability (**A**,**B**), Relative solvent accessibility (**C**), distance from mutation site to NAD+ (**D**), change of NAD+ binding affinity (**E**), and the Mutation Tolerance Ratio 2 (MTR2) (**F**) and between non-cancer diseases and benign mutations. Relative feature importance of the non-cancer pathogenic machine learning model was presented (**G**).

As identified above, the change in protein stability and the MTR score were key differentiating features in the non-cancer developmental disorders. Five features were selected in our final model including the conservation scores from PSSM, a biological score RUSR970103 [20], the atomic distance pattern from graph-based signature, the change of aromatic pharmacophore, and the neighbour frequency of short charged or polar amino acids (D and N) (Figure 6). Most features in the non-cancer pathogenic model emphasise the importance of conservation, which is largely related to the functionality of ALDHs.

## 3. Discussion

In this work, we present several potential molecular drivers of human ALDHs leading to diseases such as the change of protein stability, the conservation changes, and the residue environment from the mutation site via our mutational analysis pipeline. This was particularly evident in the non-cancer-related pathogenicity. Various computational biophysical measurement tools offered considerable contributions to this work and both the qualitative analysis and machine learning algorithms were necessary approaches to explore the links between these biochemical features and the disease-causing phenotypes. 

ALDH is crucial for aldehyde metabolism and several human ALDHs, such as ALDH1 and ALDH2, are popular targets of drug development. Our works characterised the in silico prediction of change in protein stability caused by missense variants as one of the important risk factors of pathogenicity. Compared with the qualitative analyses on the change of dimeric interaction and the change of ALDH-ligand binding, the alteration on protein stability was particularly emphasised. We suspect protein stability on ALDH monomer, the fundamental property of ALDH folding, should gain more attention in the context of missense mutations, as the destabilising effect could drastically change the conformation of some important domains of ALDH, resulting in its loss of functionality. Experimental assays also proved the necessity of thermal stability of mature ALDHs [21] It has been reported to use a small-molecule drug to stabilise a mutant protein [22], and thus, we hope our work could provide new ideas on the design of new treatments.

In addition, another molecular driver, conservational changes measured by scores from both MTR and PSSM, also revealed the strong necessity of intact protein sequence and structure. The human ALDH family contains 19 isoenzymes sharing over 40% sequence identity. Some of the regions are highly conserved, such as the Cysteine in the active site for aldehyde reaction. Though we did not find a significant difference in the change of binding affinity of the substrates, we still strongly suggest that the alteration of conserved regions may have a deleterious effect on the functions of ALDHs and both cancer and non-cancer pathogenic phenotypes.

We noticed the difference in the characterisation of cancer and non-cancer pathogenic variants from both qualitative analysis and the performance of two supervised learning models. The development of cancer is complex and heterogeneous, and for ALDHs, these proteins may contribute more to the oxidation stress during tumour growth instead of the oncogenesis. However, there is more evidence of the causation of mutated ALDHs in other non-cancer disorders. Our results also supported these findings.

One of the limitations of this work is the availability of high-quality mutation data in human ALDHs. While we initially curated over 6000 mutations from the cancerous resources, gnomAD, and ClinVar, the final dataset with several filtering criteria only contained hundreds of data points. Compared with the full dataset, our filtered dataset provided us with higher confidence in the labelling of the mutations. We also attempted to implement the same analysis process on the full dataset, but we failed to notice some distinguishable patterns between disease-causing and benign variants, especially between the cancer-risk and benign ones. This could be because of the noise (contradictory labelling of phenotypes) in the full dataset. However, when using the filtered dataset with low-quantity but high-quality data, a clearer pattern was noticed through both statistical analysis and the use of machine learning approaches. Due to the limited data, we failed to gain sufficient confidence to apply our machine learning models to all possible missense mutations to human ALDHs as previous work did [18]. Nevertheless, we are still capable of using these methods to provide potential biological driving components of diseases in ALDHs, and we hope there will be experimental validation to support these findings in future research.

In conclusion, our work provides new biological insights into the pathogenic risk factors in human ALDHs using computational methods. The molecular drivers found in this work could serve as a resource for further understanding on the functions of ALDHs and the corresponding phenotypes, which could be useful for the establishment of treatment strategies.

## 4. Materials and Method

### 4.1. Data Curation

Initially, missense mutations associated with different phenotypic consequences, namely cancer, non-cancer diseases, and benign, were collected from public resources. Mutations with conflicting labels were removed to ensure their correct annotation. Only mutations mapped with the corresponding canonical sequences of ALDH proteins were selected. The final dataset was available in the Supplementary Files.

Missense mutations associated with cancer and tumours growth were curated from three databases, Catalogue of Somatic Mutations in Cancer (COSMIC v97, released November 2022, cancer.sanger.ac.uk) [23], The Cancer Genome Atlas Program (TCGA, v37 released January 2023) [24], and cBioPortal (v5.3.3) [25,26]. To improve the quality of the labelling, we applied different filters to remove noise data. Mutations in COSMIC were filtered based on confirmed somatic status, clear sample type, and known literature support. Mutations from cBioPortal were collected from the section “Curated set of non-redundant studies”. Finally, we took the overlap of all three databases to form our cancer-risk group, as they were likely to have the strongest association with the development of cancer and tumours.

Missense mutations of ALDH associated with different non-cancer diseases were collected from ClinVar [27]. Each mutation was manually confirmed with clinical diagnosis by literature search, as previously described [17,18]. These mutations were mainly related to developmental disorders and mobility defectiveness.

Population genetic variants were collected from the Genome Aggregation Database (gnomAD, v2.2.1) [28]. An allele frequency of 1% was used to filter rare missense variants [29]. Mutations with stable occurrence across populations are less likely to be pathogenic and were labelled as benign.

### 4.2. Structural Curation of ALDH

In order to capture the structural and functional consequences of the missense mutations, three structures were curated for each of the 19 human ALDHs: an apo monomer, an apo dimer, and a protein–substrate complex. All final models are available in the Supplementary Files.

(a) ALDH monomer

The apo monomer structure was generated using the latest AlphaFold2 [30] with a template date of *2022-03-02*, as not all human ALDHs had available experimental structures. We have previously shown that AlphaFold2 models are as reliable as experimental structures for predicting the effects of missense variants [31]. The model with the highest confidence score (predicted Local Distance Difference Test, pLDDT [32]) was selected for analysis. The AlphaFold2 models were aligned with the available experimental ALDH structures, and the root mean squared deviation (RMSD) was low (Table S1).

(b) ALDH dimer

ALDH proteins typically function in larger oligomeric structures, often homotetramer or homooctamer [33]. To characterise the effects of mutations on these key protein–protein interactions, we used AlphaFold2-multimer [34] to generate the ALDH dimer with the same parameter settings and selection criteria described above. As the ALDH tetramer usually is a symmetric dimer of dimer [33], the dimeric form was used to provide useful biological insight without significant increases in required computational resources.

Human ALDHs are also suspected to play a role in the PPI network [35,36]. However, we focused more on the interaction between monomers. The interaction between ALDH and other regulatory factors could be studied in future works.

(c) ALDH with substrate binding

Despite the multiple functions of some ALDH proteins, human ALDHs play a major role in the metabolism of different aldehydes. The oxidation process is NAD(P)+ dependent, and different ALDH isoenzymes have their own preferred aldehyde molecules dictated by the size and shape of their substrate binding pocket [2,4], except ALDH16A1 which is a pseudoenzyme [13]. We, therefore, used AutoDock Vina (v1.1.2) [37] to model the NAD+ and two aliphatic aldehydes of different sizes within each ALDH structure. Docking was guided by the experimentally determined complex of ALDH2 with NAD+ (PDB ID: 1O00, chain A [38]) and a competitive inhibitor, 2P3 (PDB ID: 5L13, chain A [39]). Our docking protocol was first used to redock the ligands back to ALDH2 with high fidelity, before being applied to the AlphaFold2 models of the 18 active human ALDHs.

### 4.3. Biochemical and Functional Annotations on the Missense Mutations in ALDHs

We utilised different databases of biochemical properties and computational biophysical measurements to annotate the functional changes of missense mutations in ALDHs. All the categorical features were transformed by one-hot-encoding.

(a) Physicochemical and biochemical properties

To describe the attributes of the wild-type (WT) and mutant amino acid, each amino acid was assigned to one of the five groups based on properties of its side chain, namely hydrophobic (A, F, I, L, M, V, W, and Y), polar (N, Q, S, and T), negative charged (D and E), positive charged (H, K, and R), and special (C, G, and P). The isoelectric point (pI), molecular weight, and molecular volume [40,41] of 20 amino acids were also included for each WT and mutant residue.

We extracted different scores from multiple amino acid substitution matrices and the statistical interpretations of protein contact potentials from the biochemical databases, AAindex 2 and 3 [42]. To evaluate the conservation-based changes caused by mutations, homologous sequences of each ALDH protein were searched against the *nr* databases [43] with three iterations using PSI-BLAST [44,45] to generate the position-specific scoring matrix (PSSM). Scores were retrieved from these PSSM profiles according to the WT and mutant amino acids.

We also considered the evolution-based conservation changes by incorporating the calculations from the Missense Tolerance Ratio-viewer (MTR-viewer) website [46]. Scores from MTR2 with 31 codons window were included to assess the evolutionary pressure of purifying selection of the corresponding mutation site.

(b) Computational biophysical measurements

Missense mutation could have dramatic effect on protein folding, protein thermodynamic stability, and their interactions. Thus, we employed a number of *in-silico* biophysical methods to capture the subtle difference in ALDH structures caused by mutations. All the calculations were measured using ΔΔG (Kcal/mol), with zero as a cutoff (ΔΔG > 0: stabilising; ΔΔG < 0: destabilising).

Several tools to calculate the change of protein stability upon mutations were introduced to measure how mutations altered protein foldings. Structure-based calculations were generated by mCSM-Stability [47], SDM [48], DUET [49], ENCoM [50], DynaMut1 [51], DynaMut2 [52], and DDMut [53], while the sequence-based predictions were from the SAAFEC-SEQ [54]. Recommended parameter settings were used according to the documents of these tools. Since the AlphaFold2 protein models usually have a low confidence score in the loop region, the pLDDT was also included as one of the features to represent the disorders of the structure. The mutation effects on protein dynamics were retrieved from the Δvibrational entropy from DynaMut1. 

Oligomerization of ALDH is also one of the essential properties to its functions. In our AlphaFold2 dimer, we picked the monomer A and based our calculations on it because of the symmetry of the ALDH oligomer. We first calculated the distance of all WT residues from our target monomer to the PPI interface to annotate the position of these residues. Then we selected all mutation locus within 10 Å of the PPI interface and calculated the change of protein binding affinity caused by mutations using mCSM-PPI1 [47] and mCSM-PPI2 [55]. For the rest of the mutations, we marked the change of binding affinity as 0 to indicate little effects. 

The aldehyde oxidation metabolism is the major function of ALDH, which is strongly reliant on its intact binding to both the aldehyde substrates and NAD+ cofactor. Similarly, we initially scanned through the distance of all WT residues to these two ligands, respectively to describe their positions, followed by using mmCSM-lig [56] to evaluate the change of ligand binding affinity on those residues within 10 Å of the small molecules. The affinity changes of the rest of the mutations were marked as 0 to indicate little effects.

(c) Residue environment

The biochemical environment is crucial to justify the fitness of residue substitutions. Mutations in an unfavourable sequenced and structural environment could cause strong deleterious impact to the protein functions. 

To determine the sequence-based residue environment, the neighbour amino acid frequency in a fifteen-residue window was calculated [57]. All 20 amino acids were divided into different groups based on previous works [58] including hydrophobic (A, L, and M), aliphatic (I and V), aromatic (F, Y, and W), long polar (E, Q, K, and R), short polar (H, S, T, and C), short charged/polar (D and N), and structure-breaking (G and P). We selected the seven leading and following residues from the mutation site and the number of occurrence of a specific group of amino acids was first computed, which was further divided by the length of the ALDH sequence to generate the frequency. 

To model the structural environment at the mutation site, features were introduced from three aspects. First, some basic structural environment descriptions were included. Relative solvent accessibility (RSA) and Residue depth were computed via Biopython [59] to quantify solvent exposure. Secondary structure and torsion angles of peptide bonds (phi and psi) were calculated using the DSSP program [60,61]. Second, the residue interaction contacts of both WT and mutant structures (generated by MODELLER [62,63,64]) were computed via the Arpeggio packages [65]. Third, a number of distance patterns of atom pairs were generated by our graph-based signatures [47]. In graph-based signatures, atoms with eight different pharmacophores are considered as nodes and their contacts within a certain distance cutoff are considered as edges. The residue environment is modelled in a cumulative distribution of different types of atomic pairs through different configurations of distance steps and cutoffs from the mutation site.

(d) Functional prediction

Three conventional variant effect predictors, namely Sorting Intolerant from Tolerant (SIFT) [66], PolyPhen2 [67], and SNAP2 [68], were used to estimate the functional consequences caused by missense mutations of ALDHs.

### 4.4. Qualitative Analysis

After obtaining the annotations of mutations in ALDH from various aspects, we compared the features from computational biophysical measurements and the basic structural environment descriptions to delineate the molecular driver of cancer and non-cancer diseases. The two-tailed wilcoxon signed-rank test was used to have two binary comparisons of the means of those features, cancer vs. benign and non-cancer diseases vs. benign, respectively. Features were considered as potential molecular drivers when the statistical test presented significant differences (*p* value < 0.05).

### 4.5. Machine Learning Analysis

After the statistical comparisons of the annotations of mutations in ALDH, machine-learning analyses were deployed to investigate the link between these features and the pathogenic consequences of the mutations. Several methods of dimensionality reduction (unsupervised learning) were used to help visualise the data and clustering. Further to that, different supervised machine learning algorithms were used to study the combination of different features.

(a) Dimensionality reduction

Three methods to reduce the dimension of the datasets, namely Principal Component Analysis (PCA), t-Distributed Stochastic Neighbour Embedding (t-SNE), and Uniform Manifold Approximation and Projection (UMAP) were employed to visualise the distribution of samples of cancer, non-cancer diseases, and benign. PCA linearly transforms features to maximise the variance in a new dimension, while tSNE and UMAP are both sophisticated methods by maintaining the relationship of data points in a high dimension to a lower dimension. All these methods were performed using the R languages (R version 4.2.3) with different packages, *Rtsne* (version 0.16) [69] and *umap* (version 0.2.10) [70]. Data points on the scatter plot were coloured according to their labels.

(b) Supervised machine learning

Supervised machine learning algorithms were used to investigate the link between the biochemical and functional annotations of mutations with pathogenic and benign labels. We applied the same analysis pipeline to this work, which has been successfully used in the characterisation of the effects of mutation on other proteins [16,17,18].

In brief, a number of machine learning algorithms were tested and the one with the best predictive performance was chosen for further optimisation. Then, a greedy feature selection method was applied to establish the best combination of features and avoid overfitting, which has been explained in previous works [52,71]. In this work, we did not split the dataset into the training and blind test sets due to a relatively small sample size. However, we applied 10-fold Cross Validation (10-CV), Jack-Knife validation, and Leave-one-protein-out (LOPO) to examine the generalisability of the machine learning models. Predictive performance was evaluated by a number of metrics including balanced accuracy (BACC), F1-score, Matthew’s Correlation Coefficient (MCC), and Area Under the Receiver Operating Characteristic Curve (AUROC). Performance of the models was further benchmarked with the state-of-the-art methods including the conventional variant effect predictors (SIFT, PolyPhen2, and SNAP2) as well as the deep mutational scanning (DMS)-based methods (Envision [72] and DeMaSk [73]).

## Figures and Tables

**Table 1:** Distribution of variants of different functional consequences of human ALDHs.

Gene Name	#Benign	#Cancer-Risk	#Non-Cancer Diseases	
ALDH1A1	0	4	0	4
ALDH1A2	1	8	4	13
ALDH1A3	1	2	6	9
ALDH1B1	4	2	0	6
ALDH1L1	0	1	0	1
ALDH1L2	0	0	0	0
ALDH2	1	2	0	3
ALDH3A1	2	4	0	6
ALDH3A2	0	0	0	0
ALDH3B1	1	0	0	1
ALDH3B2	5	3	0	8
ALDH4A1	5	0	0	5
ALDH5A1	4	0	5	9
ALDH6A1	0	1	3	4
ALDH7A1	1	1	9	11
ALDH8A1	1	6	0	7
ALDH9A1	0	0	0	0
ALDH16A1	2	4	0	6
ALDH18A1	2	2	11	15
	30	40	38	108

**Table 2:** Predictive performance on identifying variants of cancer-risk in human ALDHs.

Method	Test Type	BACC	F1 Score	MCC	Recall	Precision	AUROC
Cancer-risk model	10-CV	0.777	0.814	0.558	0.828	0.802	0.785
Cancer-risk model	Jack-Knife	0.775	0.737	0.559	0.850	0.791	0.782
Cancer-risk model	Leave-one-protein-out	0.796	0.767	0.592	0.825	0.825	0.808
SIFT	/	0.675	0.693	0.346	0.650	0.743	0.784
PolyPhen2 (HumDiv)	/	0.771	0.795	0.538	0.775	0.816	0.858
PolyPhen2 (HumVar)	/	0.758	0.779	0.512	0.750	0.811	0.845
SNAP2	/	0.600	0.580	0.201	0.5	0.690	0.603
Envision	/	0.771	0.824	0.559	0.875	0.778	0.793
DeMaSk	/	0.746	0.725	0.494	0.625	0.862	0.771

**Table 3:** Predictive performance on identifying variants of non-cancer diseases in human ALDHs.

Method	Test Type	BACC	F1 Score	MCC	Recall	Precision	AUROC
Non-cancer disease model	10-CV	0.878	0.899	0.765	0.929	0.872	0.912
Non-cancer disease model	Jack-Knife	0.907	0.897	0.821	0.947	0.900	0.932
Non-cancer disease model	Leave-one-protein-out	0.884	0.871	0.764	0.868	0.917	0.931
SIFT	/	0.613	0.597	0.227	0.526	0.690	0.72
PolyPhen2 (HumDiv)	/	0.791	0.816	0.582	0.816	0.816	0.914
PolyPhen2 (HumVar)	/	0.791	0.816	0.582	0.816	0.816	0.908
SNAP2	/	0.718	0.747	0.436	0.737	0.757	0.793
Envision	/	0.807	0.857	0.652	0.947	0.783	0.843
DeMaSk	/	0.841	0.849	0.678	0.816	0.886	0.882

## Data Availability

Data is available at the Supplementary Files.

## Supplementary Materials

The supporting information can be downloaded at https://www.mdpi.com/article/10.3390/ijms241210157/s1.
