# Supplementary material for: Identifying the Molecular Drivers of Pathogenic Aldehyde Dehydrogenase Missense Mutations in Cancer and Non-Cancer Diseases
Source: Int J Mol Sci. 2023 Jun 15;24(12):10157. doi: 10.3390/ijms241210157 (PMC10299257; doi:10.3390/ijms241210157)
Supplement: Supplementary file 1 [file ijms-24-10157-s001.zip › ALDH_Supplemental_Materials.pdf]

Supporting Information

Identifying the Molecular Drivers of Pathogenic Aldehyde Dehydrogenase Missense Mutations in Cancer and Non-Cancer Diseases

Dana Jessen-Howard [1,†], Qisheng Pan [1,2,†] and David B. Ascher [1,2,*]

[1] School of Chemistry and Molecular Bioscience, University of Queensland, Brisbane, QLD 4072, Australia; d.jessenhoward@uq.net.au (D.J.-H.); qisheng.pan@uq.net.au (Q.P.)
[2] Computational Biology and Clinical Informatics, Baker Heart and Diabetes Institute, Melbourne, VIC 3004, Australia
* Correspondence: d.ascher@uq.edu.au; Tel.: +61-7-336-53991
† These authors contributed equally to this work.

TABLES

Table S1. Structural difference between AlphaFold2 models and experimental structures of human ALDHs.

Gene name	Length	PDB ID (chain A)	Canonical sequence coverage by structure	Aldehyde catalytic domain	RMSD (Å)
ALDH1A1	501	4WB9	9-501	Yes	0.449
ALDH1A2	518	4X2Q	26-518	Yes	0.600
ALDH1A3	512	6S6W	23-508	Yes	0.701
ALDH1B1	517	7MJC	25-517	Yes	0.385
ALDH1L1	902	2CFI	1-306	No	1.628
ALDH1L2	923	/	/	/	/
ALDH2	517	1O00	24-517	Yes	0.214
ALDH3A1	453	3SZA	2-448	Yes	0.230
ALDH3A2	485	4QGK	1-460	Yes	0.306
ALDH3B1	468	/	/	/	/
ALDH3B2	385	/	/	/	/

ALDH4A1	563	3V9G	23-563	Yes	0.261
ALDH5A1	535	2W8N	56-535	Yes	0.267
ALDH6A1	535	/	/	/	/
ALDH7A1	539	2J6L	31-527	Yes	0.222
ALDH8A1	487	/	/	/	/
ALDH9A1	494	6QAK	1-494	Yes	1.081
ALDH16A1	802	/	/	/	/
ALDH18A1	795	2H5G	361-793	Yes	0.392

FIGURES

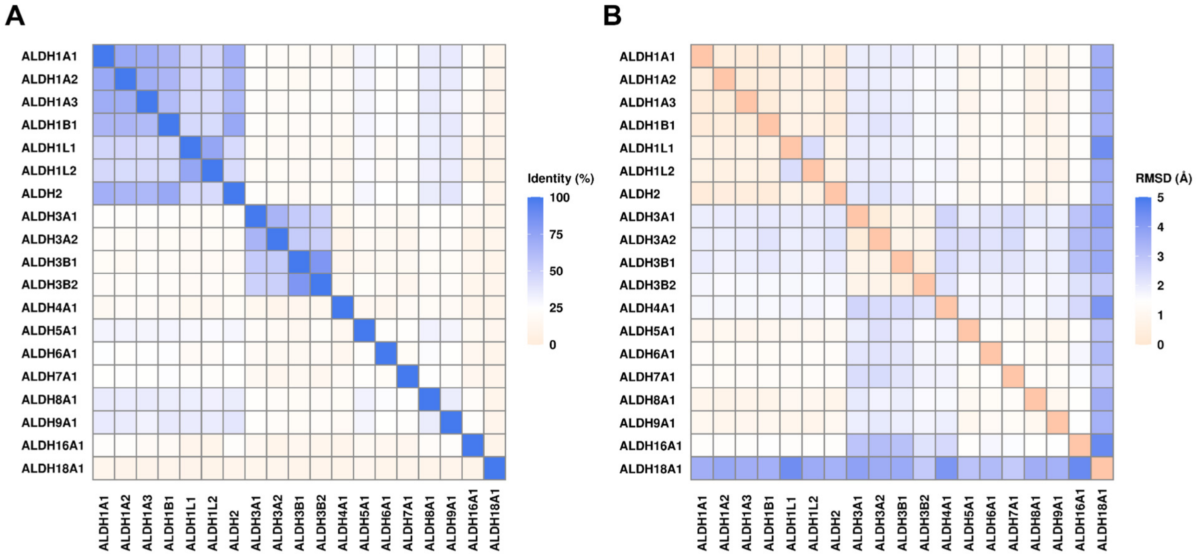

Figure S1. Sequence identity (A) and Structure similarity measured by root mean squared deviation (RMSD) (B) of 19 human ALDHs. All sequences were in canonical form. ALDH monomeric structures were generated using AlphaFold2.

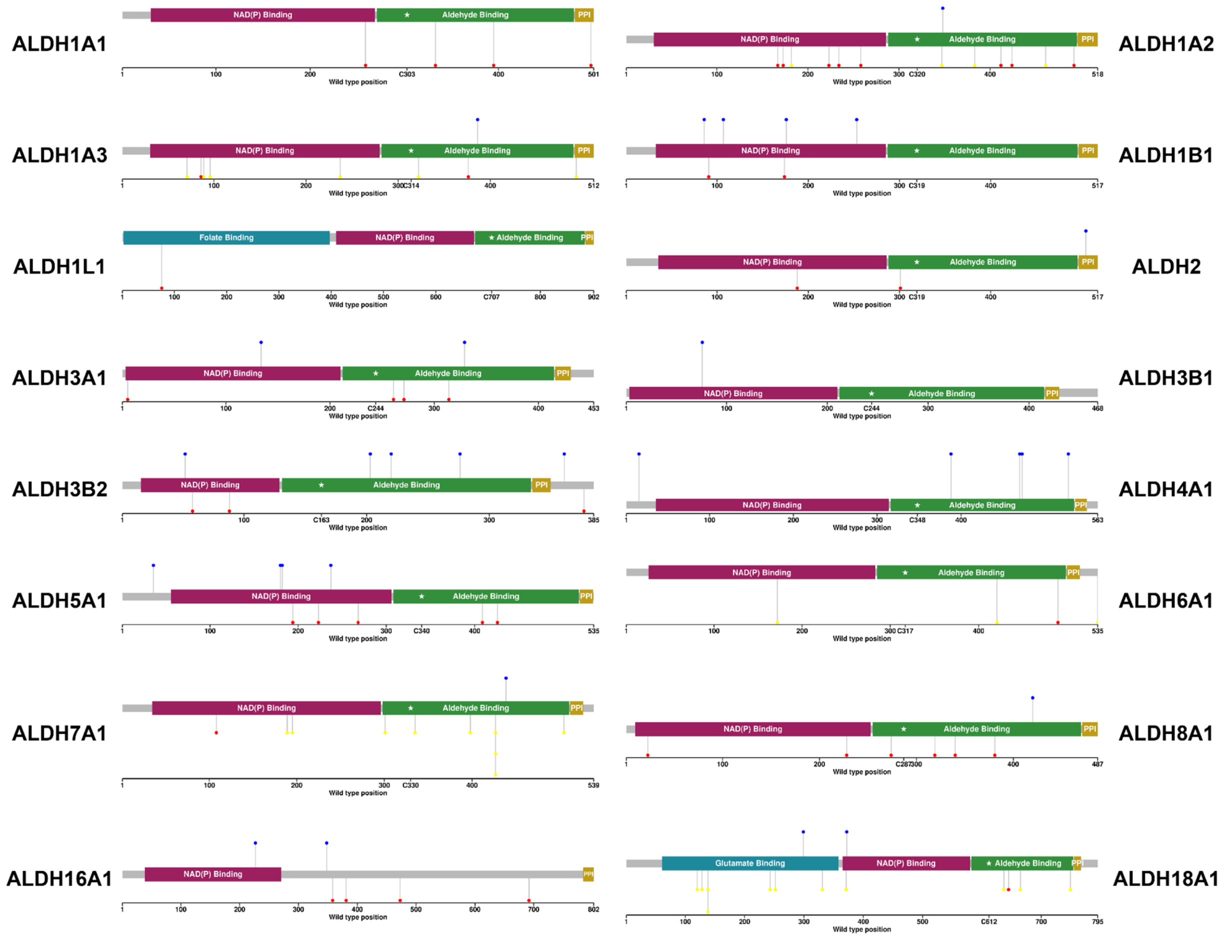

Figure S2. Distributions of variants of three labels, namely cancer-risk (red), benign (blue), and non-cancer diseases (yellow) of the sequences of human ALDHs. Each ALDH protein is coloured based on its different important regions, namely NAD(P)+ binding region (dark magenta), aldehyde binding region (dark green), protein-protein interaction region (dark yellow), and addition domains such as folate/glutamate binding region (dark cyan).

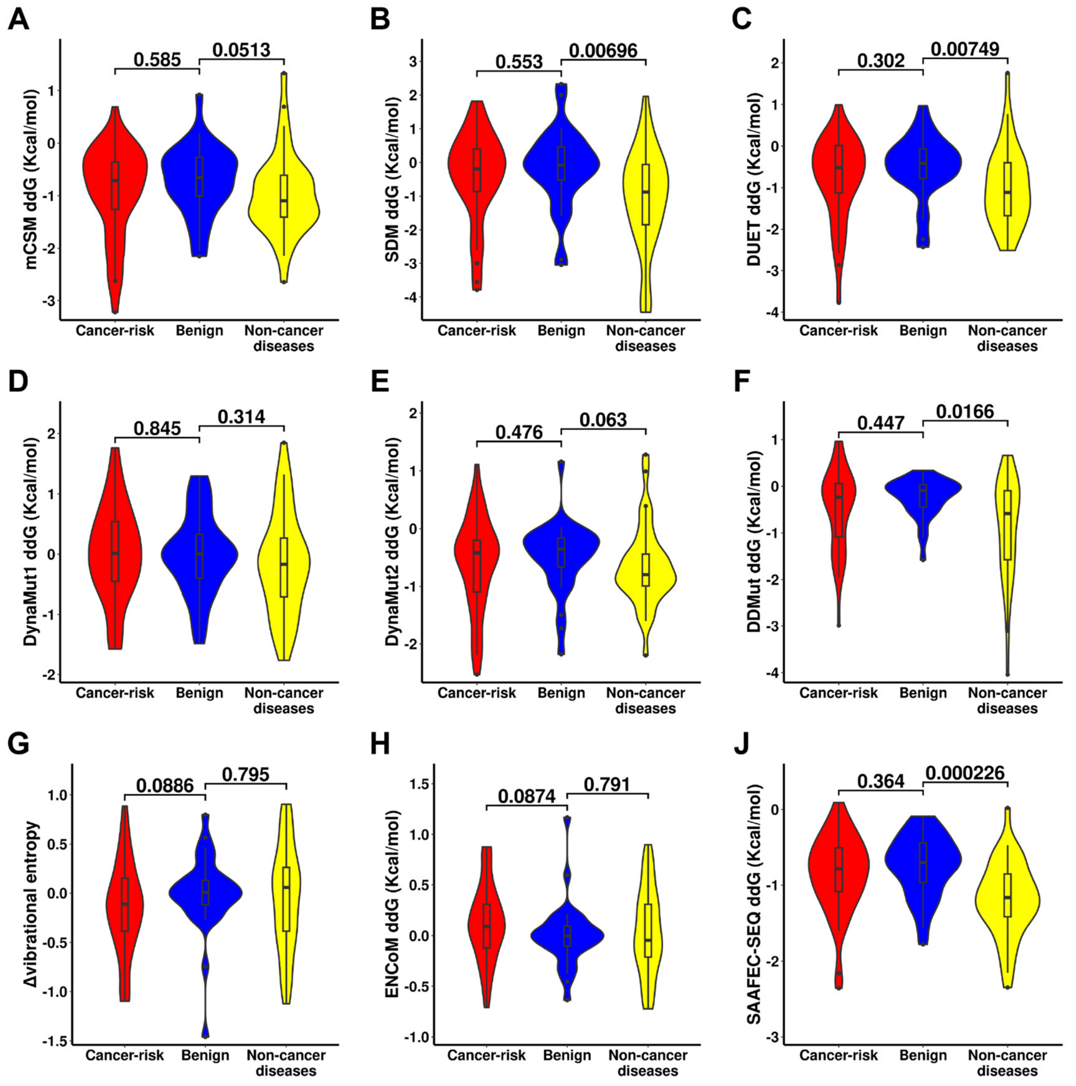

Figure S3. Qualitative comparison of the change of protein stability of "cancer *vs* benign mutations" and "non-cancer diseases *vs* benign mutations" in human ALDHs. Wilcox signed-rank test was used and p-value was presented above the bracket of two comparisons, respectively (significance level: $p < 0.05$).

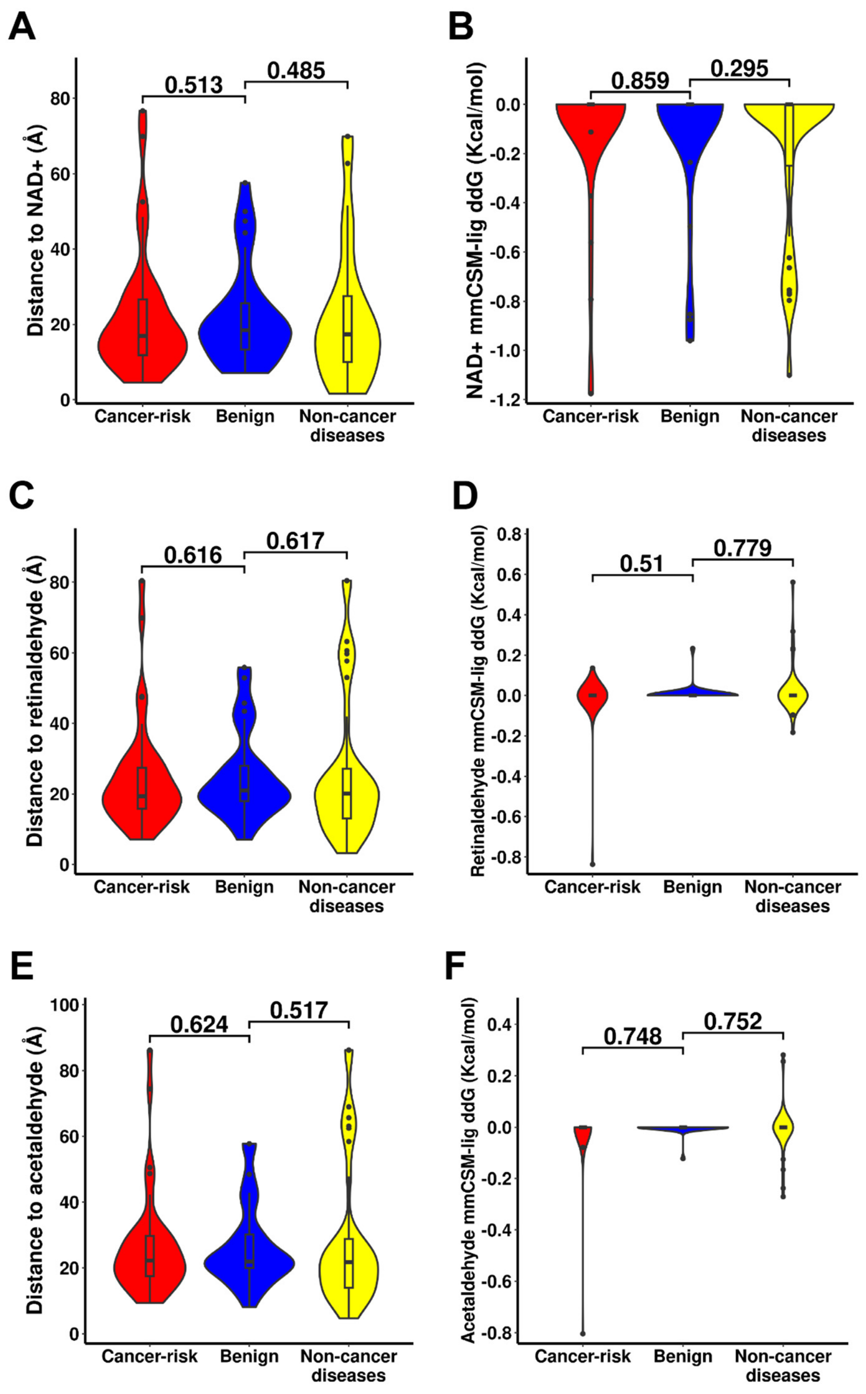

Figure S4. Qualitative comparison of the change of ligand binding affinity of "cancer *vs* benign mutations" and "non-cancer diseases *vs* benign mutations" in human ALDHs. The distance from the mutation site and the change of binding affinity to three substrates, namely NAD+ (A-B), retinaldehyde (C-D), and acetaldehyde (E-F) were presented, respectively. Wilcox signed-rank test was used and p-value was presented above the bracket of two comparisons, respectively (significance level: $p < 0.05$).

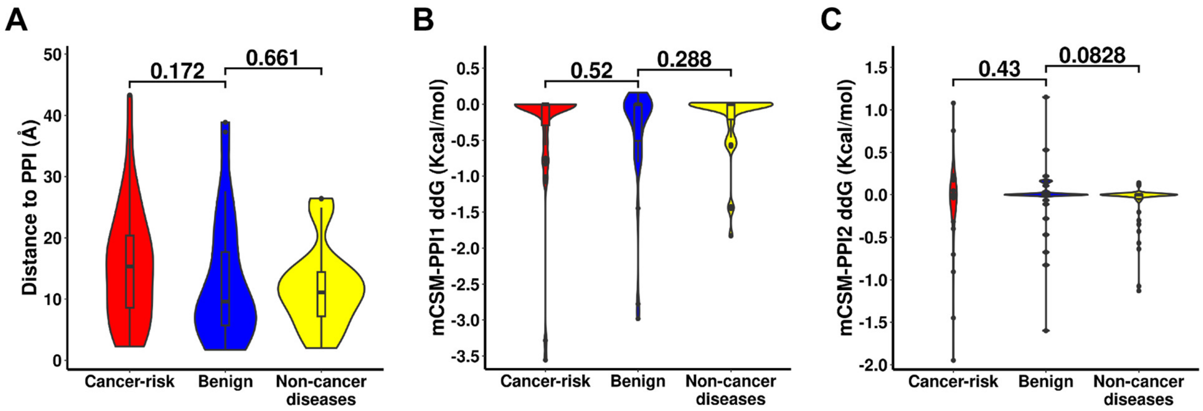

Figure S5. Qualitative comparison of the change of protein-protein interaction (PPI) affinity of "cancer *vs* benign mutations" and "non-cancer diseases *vs* benign mutations" in human ALDHs dimer. Both the distance to PPI interface (A) and the change of binding affinity (B-C) were presented. Wilcox signed-rank test was used and p-value was presented above the bracket of two comparisons, respectively (significance level: $p < 0.05$).

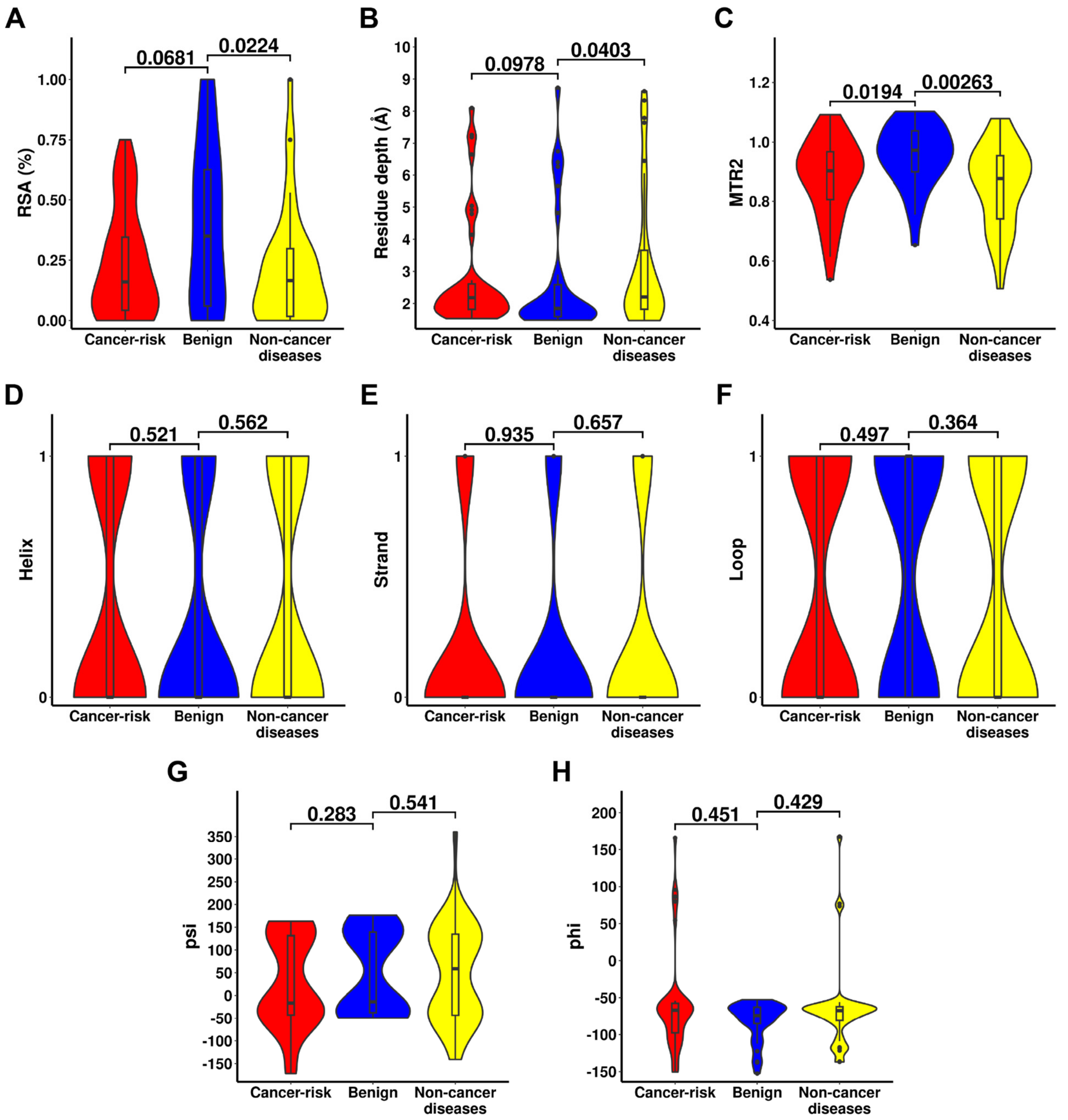

Figure S6. Qualitative comparison of the residue environment at the mutation site, including relative solvent accessibility (RSA) (A), residue depth (B), Mutation Tolerance Ratio 2 (MTR2) score (C), secondary structure type (D-F), and dihedral angles (G-H) of "cancer *vs* benign mutations" and "non-cancer diseases *vs* benign mutations" in human ALDHs. Wilcox signed-rank test was used and p-value was presented above the bracket of two comparisons, respectively (significance level: $p < 0.05$).

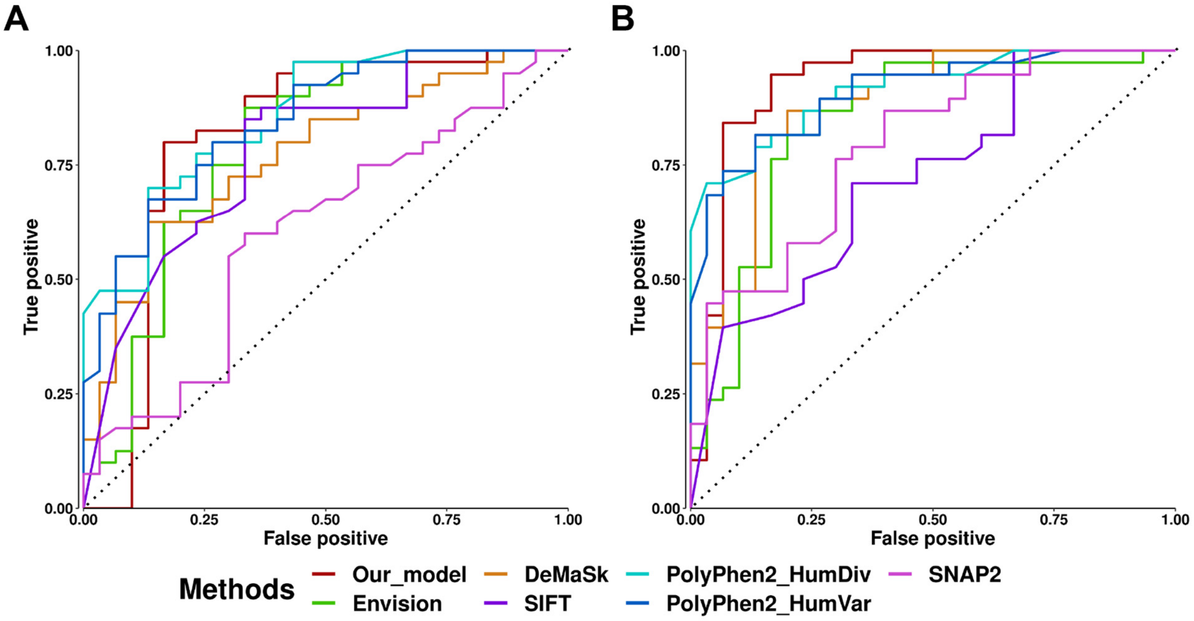

Figure S7. Performance comparison between our cancer-risk model (A) and non-cancer pathogenic model (B) and the state-of-the-art variant effect predictors.
